# Autophagy-related IFNG is a prognostic and immunochemotherapeutic biomarker of COAD patients

**DOI:** 10.3389/fimmu.2023.1064704

**Published:** 2023-01-23

**Authors:** Taohua Yue, Yunlong Cai, Jing Zhu, Yucun Liu, Shanwen Chen, Pengyuan Wang, Long Rong

**Affiliations:** ^1^ Department of Endoscopy Center, Peking University First Hospital, Peking University, Beijing, China; ^2^ Division of General Surgery, Peking University First Hospital, Peking University, Beijing, China

**Keywords:** colon adenocarcinoma (COAD), autophagy, IFNg, prognosis, immunotherapy, cisplatin

## Abstract

**Background:**

Numerous studies have shown autophagy affects cellular immune responses. This study aims to explore prognosis and immunotherapeutic biomarkers related to autophagy in colon adenocarcinoma (COAD).

**Methods:**

Based on R software, we performed the ssGSEA, differential expression analysis, Kaplan-Meier survival analysis, correlation analysis, and enrichment analysis. For wet experiment, we did qRT-PCR, immunohistochemistry and CCK-8 experiments.

**Results:**

Using autophagy-related genes (ARGs) and the ssGSEA, COAD patients were divided into low and high autophagy groups. For immune score, stromal score, tumor purity, tumor infiltrating immune cells, co-signaling molecules, tumor mutational burden, microsatellite instability, mismatch repair, immune-related pathways, immune signatures, somatic mutations and subtype analysis, high autophagy group might benefit more from immunotherapy. Among 232 ARGs, IFNG was generally significantly correlated with tumor immunotherapy biomarkers (PD-L1, CD8A and cytotoxic T lymphocytes (CTL)). The disease-free survival of high IFNG group was significantly longer than that of low group. On above-mentioned immune-related research, the high IFNG group reached the same conclusion. The qRT-PCR and IHC analysis confirmed that IFNG was significantly higher expressed in dMMR samples compared to pMMR samples. For chemotherapy, the autophagy and IFNG were significantly negatively related to the chemosensitivity to cisplatin; IFNG inhibitor glucosamine increased cisplatin chemoresistance while IFNG increased cisplatin chemosensitivity; IFNG could reverse glucosamine induced chemoresistance. The functional enrichment analysis of IFNG, PD-L1, CD8A and 20 similar proteins were related to the activation of the immune system. The GSEA and ceRNA network partly described interaction mechanisms of IFNG with PD-L1 and CD8A.

**Conclusion:**

Autophagy score and IFNG expression were novel immunotherapy predictive biomarkers, which might play predictive effects through the JAK-STAT signaling pathway. IFNG might be a potential targeted therapy for cisplatin resistant colon cancer. Besides, IFNG was also a prognostic indicator.

## Introduction

According to the Global Cancer Statistics 2022, colon cancer (CC) is the second leading cause of cancer-related deaths worldwide, whose incidence ranks third (1). Colon adenocarcinoma (COAD) is the most common type of primary CC. Although the American Joint Committee on Cancer (AJCC) classification can be used to assess the prognosis of COAD patients, overall survival (OS) and disease-free survival (RFS) is not always associate with tumor stage. Therefore, we urgently need to mine the prognostic biomarkers of COAD patients.

Complete mesocolicexcision is the standard treatment for COAD. Conventional chemotherapy, radiotherapy, targeted therapy, and anti-angiogenesis therapy are also optional treatment solutions. Immunotherapy has recently become a novel therapy for cancer treatment. However, which COAD patients are suitable for immunotherapy need to be resolved urgently.

Autophagy is the catabolic process of eukaryotic cells, which can quickly provide fuel to supply energy or provide materials to renew cell components (2). Recent studies had revealed that autophagy was closely related to the occurrence and development of tumors, and it played a dual role in suppressing or promoting cancer. Besides, autophagy participated in innate immunity, inflammatory response (3) and adaptive immune response by processing antigens (4) and regulating the development and function of lymphocytes (5). Therefore, autophagy as a molecular target for cancer therapy and its immune relevance had attracted more attention.

**Figure 1:**
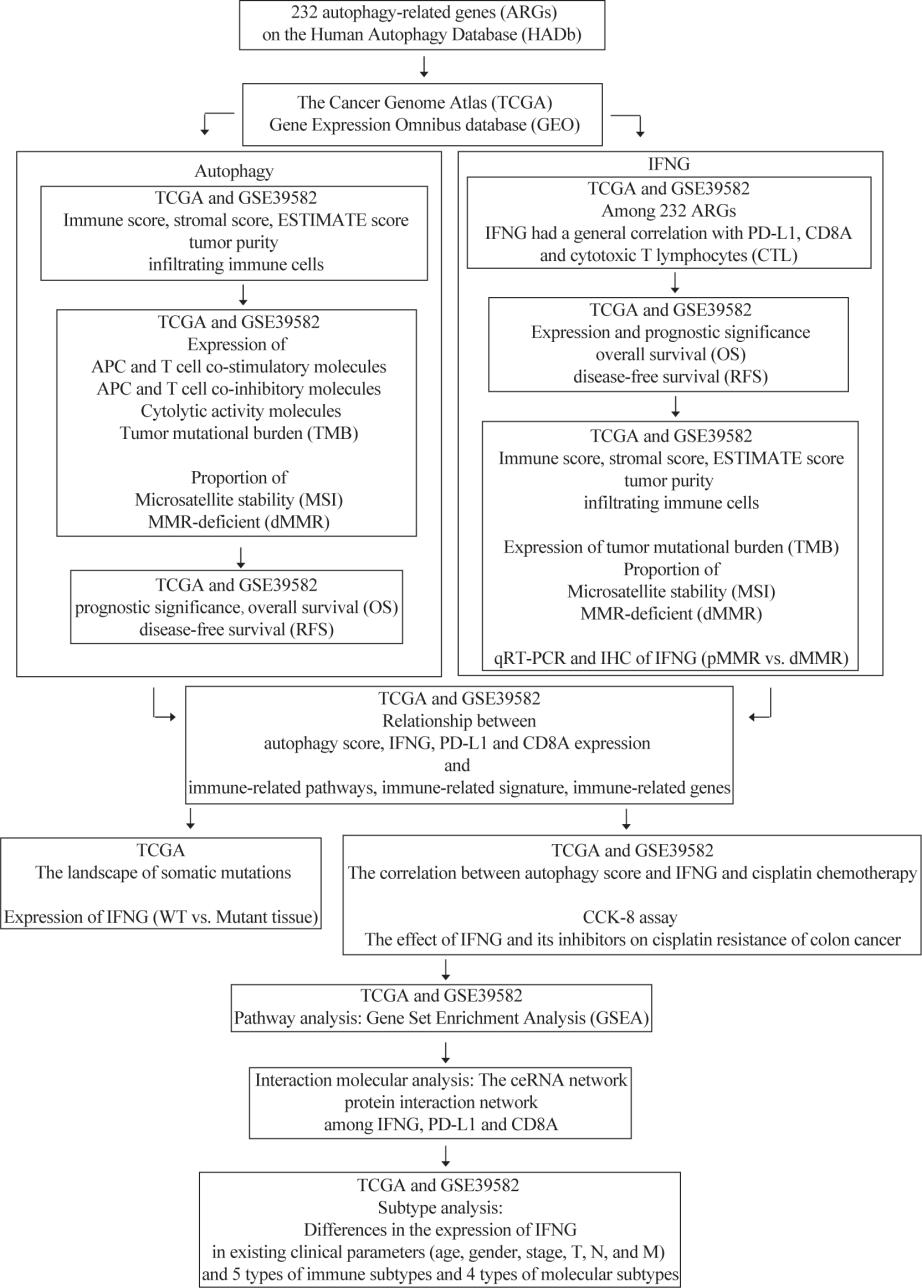
The flow chart of our research.

The flow chart of our research was displayed in **Figure 1**. In our study, combined with proven immunotherapy predictive markers, COAD of the high autophagy score group or high IFNG group tended to be “hot tumor” tissues and were more suitable for immune checkpoint inhibitor therapy. Besides, IFNG might be a potential targeted therapy for cisplatin resistant colon cancer. COAD patients of the high IFNG group had significantly longer disease-free survival (RFS).

## Materials and methods

### The TCGA and GSE39582 data

The transcriptome data and corresponding clinical information of colon adenocarcinoma (COAD) were downloaded from the TCGA (https://genome-cancer.ucsc.edu/) and GEO database (https://www.ncbi.nlm.nih.gov/geo/). For TCGA data set, fragments per kilobase of transcript per million mapped reads (FPKM) were converted to TransPerKilobase of exon model per Million mapped reads (TPM) and used in our research (6). For GSE39582 data set, log2 normalized data was used in this study (7). This study was approved by the Peking University First Hospital Biomedical Research Ethics Committee. All patients related to our study signed an informed consent agreement.

### Autophagy-related genes

On the Human Autophagy Database (HADb, http://www.autophagy.lu/), we collected 232 autophagy-related genes (ARGs) (**Table S1**).

### Stromal and immune scores of the tumor microenvironment and tumor purity

Tumor-infiltrating immune cells (TIICs) and stromal cells constitute the main part of normal cells around tumor cells, which can not only interfere with tumor signals but also play a vital role in cancer treatment and prognosis. Based on 2 gene signatures (the stromal and immune signatures) and the ESTIMATE algorithm (8), the abundance of non-tumor cells (TIICs and stromal cells) and tumor purity of COAD tissues are quantified. The stromal signature represents stromal cells, while the immune signature was designed to represent the abundance of TIICs in the TME. In our study, the results of ESTIMATE algorithm were presented as immune score, stromal score, ESTIMATE score and tumor purity. The higher the score, the greater the ratio of the corresponding component. Based on the ESTIMATE score (Sum of immune score and stromal score), tumor purity was inferred in tumor tissues.

### The single-sample gene set enrichment analysis

The ssGSEA is an extension of GSEA and calculates a separate enrichment score for each sample. Each ssGSEA enrichment score represents the degree to which the genes in a particular gene set are coordinately up-or down-regulated within a sample (9). In our study, based on232 ARGs, 30 immune-related pathways (10), 10 immune signatures (11) and Gene Set Variation Analysis (GSVA) package (12), the ssGSEA score was used to calculate the autophagy score and analyze the activity of immune functions in COAD transcriptome data.

### TIDE: Tumor immune dysfunction and exclusion

The TIDE (http://tide.dfci.harvard.edu/) stands for Tumor Immune Dysfunction and Exclusion, which is a computational framework developed to evaluate the potential of tumor immune escape from the gene expression profiles of public cancer cohorts (13). Based on this website, we explored the correlation between IFNG mRNA expression and cytotoxic T lymphocyte level in various public cohorts.

### RNA isolation and quantitative reverse transcription PCR

The total RNA was isolated according to the protocol of TRIZOL reagent (Life Technologies). The mRNA expressions of IFNG and GAPDH were measured by real-time PCR system (Applied Biosystems, Carlsbad, USA). The data were obtained by normalizing IFNG gene Ct (cycle threshold) values with corresponding GAPDH Ct, and then analyzed with 2-ΔΔCt method (14). The primers sequences are as follows: IFNG forward primer (5’-TCGGTAACTGACTTGAATGTCCA-3’) and IFNG reverse primer (5’-TCGCTTCCCTGTTTTAGCTGC-3’), GAPDH forward primer (5’-GGAGCGAGATCCCTCCAAAAT-3’) and GAPDH reverse primer (5’-GGCTGTTGTCATACTTCTCATGG-3’).

### Immunohistochemical analysis

The fresh COAD specimens were immersed in 10% formalin for 24 hours, then embedded in paraffin and fixed onto slides. Citrate solution (pH 6.0) was used for antigen retrieval. After endogenous peroxidase blocking, Slides were incubated with anti-human IFNG protein antibody (1:100; Proteintech) overnight at 4°C. Finally, the DAB staining system was used to detect the target protein.

### Immunohistochemical score

Immunohistochemical score of tissue section = staining intensity score * percentage of positive cells stained. Staining intensity score: 0 for no staining, 1 for weak staining, 2 for medium staining, and 3 for strong staining. Percentage of positive cells: 0 for 0 ~ 5% positive staining, 1 for 6 ~ 25% positive staining, 2 for 26 ~ 50% positive staining, 3 for 51 ~ 74% positive staining, 4 for > 75% positive staining.

### Heat maps and correlation plots

With the help of the “pheatmap” and “corrplot” packages, we draw heat maps and correlation plots, respectively.

### The TIMER2.0 database

The Tumor IMmune Estimation Resource (TIMER) (http://cistrome.dfci.harvard.edu/TIMER/) is a friendly website for systematical evaluations of the clinical significance of TIICs in pan-cancer (15). Gene_Corr module is used to explore the correlation between interested genes with a list of genes in pan-cancer. Gene_DE module is designed to mine the differential expression between tumor and adjacent normal tissues for any gene of interest across all TCGA tumors. The abundance of TIICs was calculated by the “MCPcounter” package (16) and estimated on the 2.0 version of this database (17).

### The survival and multivariate cox analysis

In our study, according to the respective median of autophagy score, IFNG, PD-L1 and CD8A expression, COAD patients were divided into the corresponding low and high groups. With “survival” and “survminer” packages, the correlation between overalls survival (OS), disease-free survival (RFS) and autophagy score, IFNG expression were measured using Kaplan–Meier analysis and log-rank test. Multivariate cox analysis tested the independent prognostic effects of autophagy score and IFNG. *P* value < 0.05 was considered statistically significant.

### The tumor mutational burden

The TMB is defined as the total number of somatic gene coding errors, base substitutions, gene insertion or deletion errors detected per million bases. Based on the maf file, mutations of cancer patients were calculated. The TMB values represented the ratio of mutation number to exon length (18).

### Mutation waterfall charts

The R package “maftools” was used to process and visualize the somatic mutation data (19).

### Cisplatin chemosensitivity assessment

In this study, we used an R package “pRRophetic” to evaluate cisplatin chemotherapy sensitivity for the COAD patients of TCGA and GSE39582 cohorts (20) and presented it in the form of the half-maximal inhibitory concentration (IC50). With the help of the “ggpubr” package, we described the correlation between cisplatin IC50 and autophagy score, IFNG, and PD-L1 expression.

### CCK-8 assay

The CCK-8 assay was used to measure DLD-1 cell viability according to the manufacturer’s instructions. Using CCK8 assay, we tested the cisplatin resistance of previously cultured DLD-1 drug resistant cell lines. Besides, to determine whether IFNG and its inhibitors glucosamine (https://go.drugbank.com/drugs/DB01296) are the target of COAD cisplatin resistance, using cisplatin resistant DLD-1 and its IC50 (25μmol/L), we compared the survival rate between control group (treated with DMSO or PBS, glucosamine dissolved in DMSO, IFNG dissolved in PBS)、glucosamine group (1000 mg/L, Sigma-Aldrich, St Louis, MO) (21)、IFNG group (500 U/mL, Shionogi Pharmaceutical Co. Osaka, Japan) (22) and glucosamine + IFNG group.

### The GSEA

Based on 186 KEGG pathways and 50 Hallmarks on the Molecular Signatures Database (MSigDB) (http://www.gsea-msigdb.org/gsea/index.jsp) (23), the GSEA was performed to predict biological processes between low and high groups. The *P*-value < 0.05 and FDR (false discovery rate) < 0.25 were set as the cutoff.

### The ceRNA network

The miRTarBase database (http://mirtarbase.mbc.nctu.edu.tw/) is a database dedicated to collecting microRNA-mRNA targeting relationships (MTI, MicroRNA-Target Interactions) supported by experimental evidence (24). In our study, the screening criteria for miRNA-mRNA interaction were verified by the most rigorous luciferase reporter assay experiment (25). The interactions of miRNA-lncRNA were predicted by using miRanda program on the Encyclopedia of RNA Interactomes (ENCORI) (http://starbase.sysu.edu.cn/index.php) (26) and the screening criteria were set at pancancerNum > 10 and clipExpNum > 4. The ceRNA network was visualized by the Cytoscape software (27).

### The GeneMANIA database

The GeneMANIA (http://www.genemania.org) is a database similar to STRING, based on which we can find genes with similar functions of interested genes and predict gene functions simultaneously (28).

### The UCSC Xena database

The immune subtypes and molecular subtypes of TCGA cohort were downloaded on the UCSC Xena TCGA Pan-Cancer (PANCAN) (29).

### Statistical analysis

All analyses were performed with R version 4.0.3 (http://www.R-project.org). The statistical significance between two group was computed by the Wilcoxon test and annotated by the number of stars (*P-value < 0.05; **P-value <0.01; ***P-value <0.001). The differences between multiple groups were tested by the Kruskal-Wallis test and corrected using the Dunn test.

## Results

### Immune microenvironment landscape of normal and colon cancer tissues in low and high autophagy groups

**Figure 1** was the flow chart of our study. Numerous studies have shown that autophagy affects the survival, differentiation and function of antigen-presenting cells (APC) and T cells and relates to anti-tumor immunity. Based on 232 ARGs and ssGSEA algorithm, we quantified autophagy activity of colon cancer tissues.

**Figure 2:**
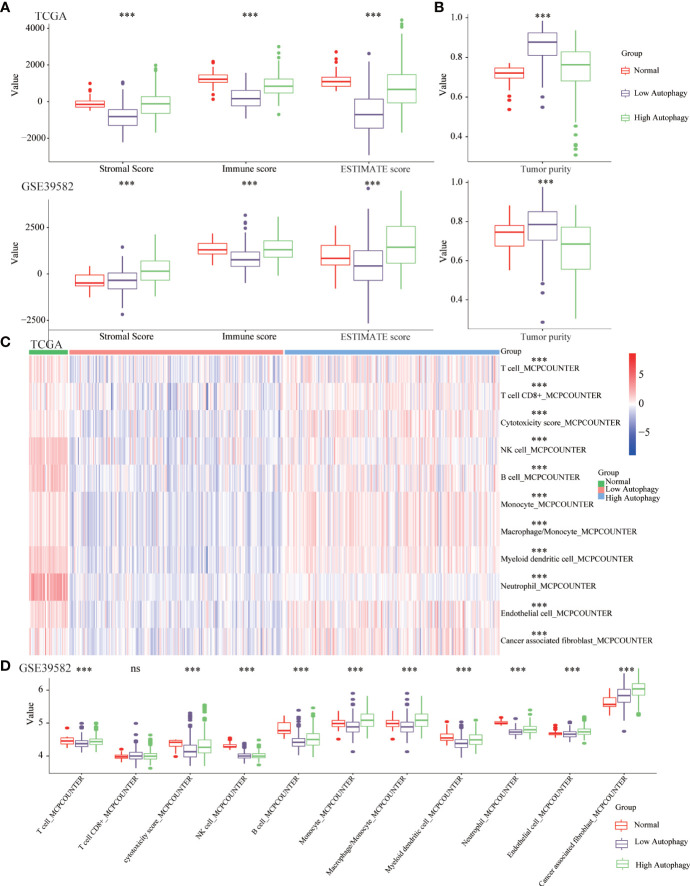
The COAD tissues of the high autophagy group were more like “hot” tumors. Comparison of the stromal score, immune score, ESTIMATE score **(A)**, tumor purity **(B)**, abundance of 11 kinds of tumor-infiltrating immune cells (TIICs) **(C, D)** between normal, low and high autophagy score group. (***P-value <0.001; ns, P-value > 0.05).

Growing evidence revealed the tumor microenvironment (TME), an aggregation of tumor cells and surrounding non-tumor cells (including tumor-infiltrating immune cells (TIICs) and stromal cells), was crucial in tumor biology. In normal tissues and high autophagy tissues, the abundance of immune and stromal components was significantly higher than that of low autophagy tissues (**Figure 2A**). Consistently, in the low autophagy group, the tumor purity, that is, the proportion of tumor cells was significantly higher than that of normal and high autophagy group (**Figure 2B**).

A large number of studies had confirmed that TIICs could affect the efficacy of immunotherapy and the prognosis of cancer patients (30, 31). In normal and high autophagy tissues, the abundance of most TIICs was significantly higher than that of low autophagy tissues (**Figures 2C, D**). In short, compared with the low autophagy group, the colon cancer tissues of the high autophagy group were more like “hot tumors” and might be more suitable for immunotherapy.

### Differences of co-signaling molecules (co-stimulatory molecules, co-inhibitory molecules), cytolytic related genes, tumor mutational burden, microsatellite instability and mismatch repair

**Figure 3:**
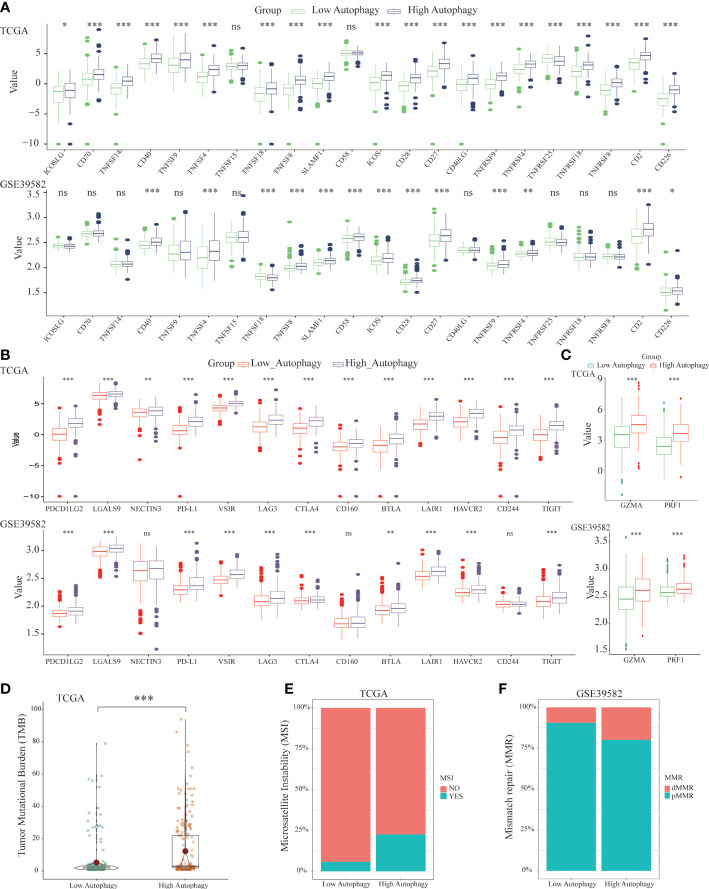
Differences of co-signaling molecules (co-stimulatory molecules, co-inhibitory molecules), cytolytic activity genes, tumor mutational burden (TMB), microsatellite instability (MSI) and mismatch repair (MMR). Comparison of the APC and T cell co- stimulatory molecules **(A)**, co-inhibitory molecules **(B)**, cytolytic related genes **(C)**, tumor mutational burden (TMB) **(D)** between low and high autophagy group. The high autophagy group had a higher proportion of MSI **(E)** and dMMR **(F)**. (*P-value < 0.05; **P-value <0.01; ***P-value <0.001).

To further explore the effect of autophagy on anti-tumor immunity, we studied the correlation between autophagy score and co-signaling molecules (11, 32). As shown in **Figure 3A**, compared with the low autophagy group, expressions of most antigen-presenting cell (APC) and T cell co-stimulatory molecules were significantly higher in the high autophagy group. Similarly, expressions of most co-inhibitory molecules were also significantly higher in the high autophagy group (**Figure 3B**). Cytolytic related genes, GZMA and PRF1, was proved to be a biomarker for antitumor immunity (33). In the high autophagy group, expressions of GZMA and PRF1 were both significantly increased (**Figure 3C**). Therefore, we speculated that COAD patients in the high autophagy group might be more suitable for immunotherapy.

Apart from the above immunotherapeutic biomarkers, TMB, MSI, and MMR were also evaluation indicators for the efficacy of immunotherapy (34, 35). Tumor tissues with a high autophagy score had more TMB (**Figure 3D**), and higher proportions of MSI (**Figure 3E**) and deficient MMR (dMMR) (**Figure 3F**). For colon cancer patients, the high autophagy group would benefit more from immunotherapy.

### The survival significance of autophagy score

In addition to the prediction of immunotherapy efficacy, we further studied the prognostic significance of autophagy score. In terms of overall survival (OS), the autophagy score had no relationship with the prognosis of COAD patients. The disease-free survival (RFS) of COAD patients in the low autophagy group was not significantly different from that of the high group, but the RFS of the low score group tended to be shortened (*P-*value was close to 0.05) (**Figure S1**).

### Autophagy-related IFNG was significantly associated with PD-L1 and CD8A

**Figure 4:**
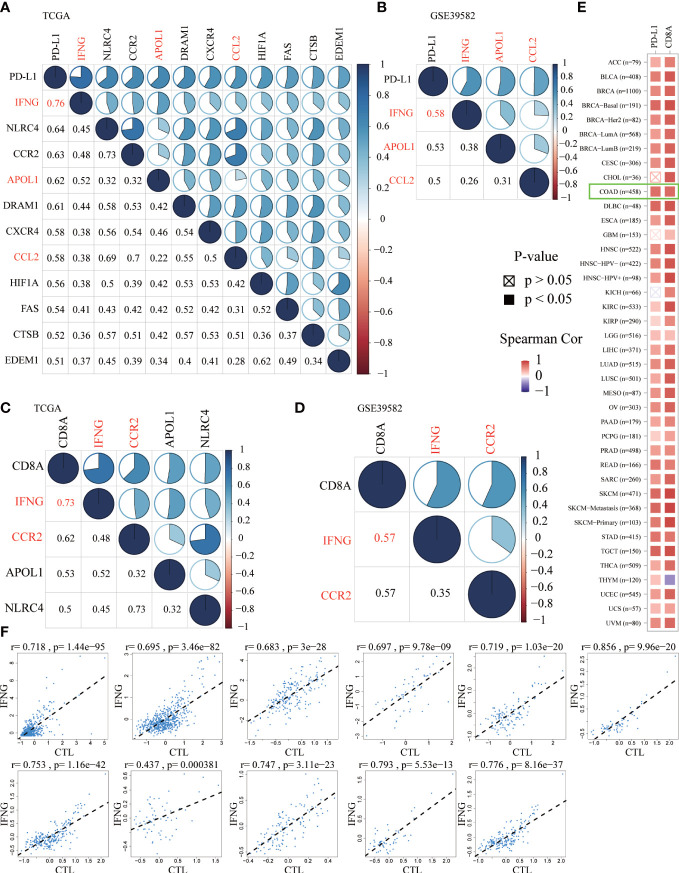
The Spearman correlation analysis of autophagy-related genes (ARGs) with PD-L1 **(A, B)** and CD8A **(C, D)** in the TCGA and GSE39582 (Spearman correlation coefficient > 0.5). The shared ARGs were marked in red. Blue represented positive correlation while red represented negative correlation. The darker the color, the greater the correlation. The numbers in the lower left corner and the pie chart in the upper right corner of the correlation graph represented the Spearman correlation coefficient in different forms. **(E)** On the TIMER2.0 website, in pan-cancer, we confirmed the general correlation between IFNG and PD-L1 and CD8A. **(F)** On the TIDE website, based on various public cohorts, IFNG expression was significantly and positively correlated with cytotoxic T lymphocyte (CTL) abundance.

Previous studies have confirmed that, in the predictive nivolumab cohort, a combination of PD-L1 and CD8A was highly predictive of progression-free survival (PFS) (36). To screen ARGs that could simultaneously predict prognosis and the efficacy of immunotherapy, the Spearman correlation analysis was performed between 232 ARGs and PD-L1 and CD8A. Based on the correlation coefficient greater than 0.5, we obtained 11 and 3 ARGs significantly related to PD-L1 in the TCGA (**Figure 4A**) and GSE39582 (**Figure 4B**), respectively. In addition, there were 4 and 2 ARGs that had a correlation coefficient greater than 0.5 with CD8A in the above 2 data sets, respectively (**Figures 4C, D**). From this, we concluded that IFNG was the ARG most significantly related to PD-L1 and CD8A. In the TIMER2.0 database, we further confirmed that in pan-cancer, IFNG had a general correlation with PD-L1 and CD8A (**Figure 4E**). Cytotoxic T lymphocytes (CTL) could specifically kill tumor cells directly and repeatedly. On the TIDE website, IFNG expression was significantly and positively associated with CTL abundance of COAD microenvironment (**Figure 4F**).

### IFNG was differentially expressed and significantly correlated with the prognosis of COAD patients

**Figure 5:**
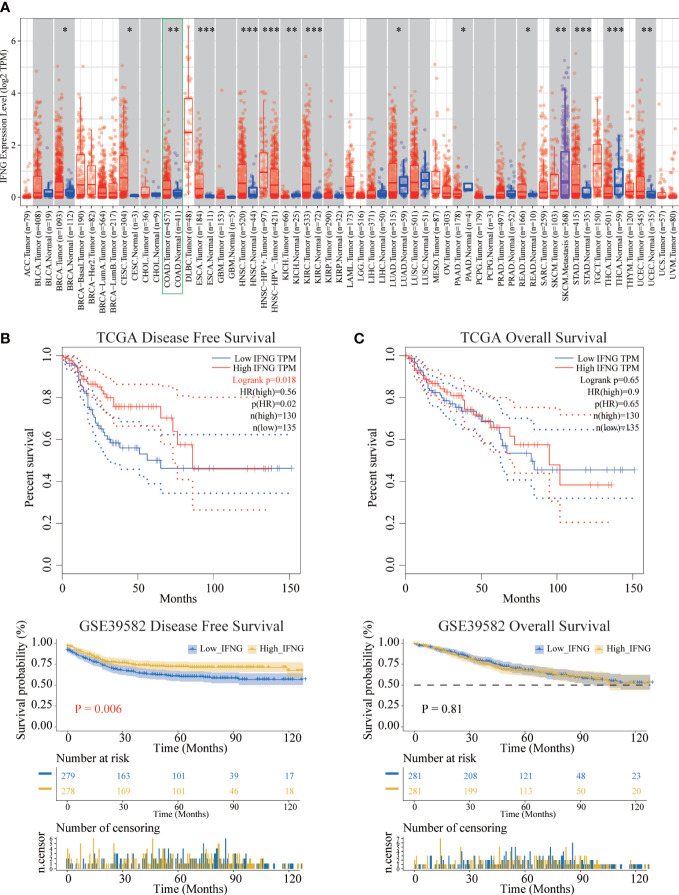
Differential expression and prognostic significance of IFNG. **(A)** In most types of TCGA pan-cancer, the expression of IFNG was significantly different between tumor and normal tissues. (*P-value < 0.05; **P-value <0.01; ***P-value <0.001). **(B)** The disease-free survival (RFS) of the high IFNG group was significantly longer than that of the low IFNG group. **(C)** There was no difference in overall survival (OS).

To explore the possible roles of IFNG in carcinogenesis, we first analyzed its expression in the TCGA pan-cancer. IFNG was significantly differentially expressed in the BRCA, CESC, COAD, ESCA, HNSC, KICH, KIRC, LUAD, PAAD, READ, SKCM, STAD, THCA and UCEC (**Figure 5A**). We speculated that IFNG might function as a crucial regulator in the carcinogenesis of the above types of cancer.

Next, survival analysis of IFNG in COAD was performed. Two prognostic indices, the RFS and OS, were included in our research. For RFS, increased expression of IFNG indicated a favorable prognosis of COAD patients (**Figure 5B**). For OS, no statistical significance of IFNG for predicting the prognosis of patients in COAD (**Figure 5C**). Multivariate cox analysis confirmed that IFNG was an independent prognostic factor for COAD patients adjusted by T, N, M and stage, while autophagy score was not an independent prognostic factor (**Figure S2**).

### Immune microenvironment landscape among normal and low and high IFNG COAD tissues

**Figure 6:**
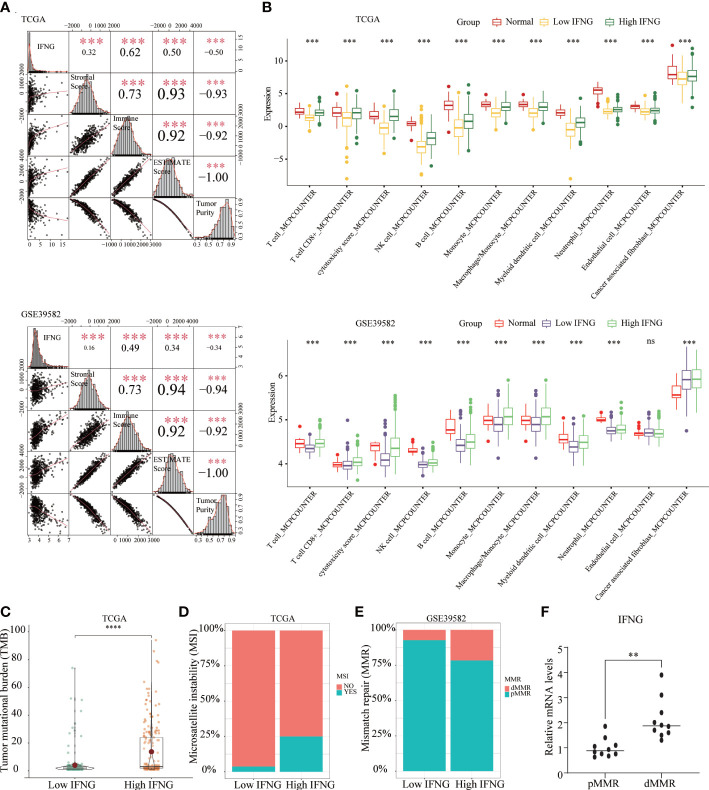
The COAD in the high IFNG group tended to be “hot tumor”, which were suitable for immunotherapy. **(A)** IFNG was significantly and positively correlated with the immune score, stromal score, and ESTIMATE score, while significantly and negatively correlated with tumor purity. **(B)** Compared with the low IFNG group, most TIICs in the high IFNG group had higher abundance, and the high IFNG group was more like normal tissues. Compared with the low IFNG group, the high IFNG group had a higher TMB **(C)** and a higher proportion of MSI **(D)** and dMMR **(E)**. (**P-value <0.01; ***P-value <0.001; ****P-value <0.0001).. **(F)** IFNG mRNA expression in pMMR and dMMR tissues.

In addition to predicting the prognosis of COAD patients, we further explored whether IFNG, like the autophagy score, could predict the efficacy of immunotherapy. Significant associations were observed between stromal, immune, ESTIMATE scores, tumor purity and IFNG expression (**Figure 6A**). In terms of immune components of the TME, the TIICs abundance of the high IFNG group was more similar to normal tissues and significantly higher than that of the low IFNG group (**Figure 6B**). Consistently, COAD tissues of the high IFNG group had higher TMB (**Figure 6C**), and more of them were microsatellite instability (MSI) (**Figure 6D**) and MMR-deficient (dMMR) (**Figure 6E**). We concluded that COAD patients of the high IFNG group might be more suitable for immunotherapy.

**Figure 7:**
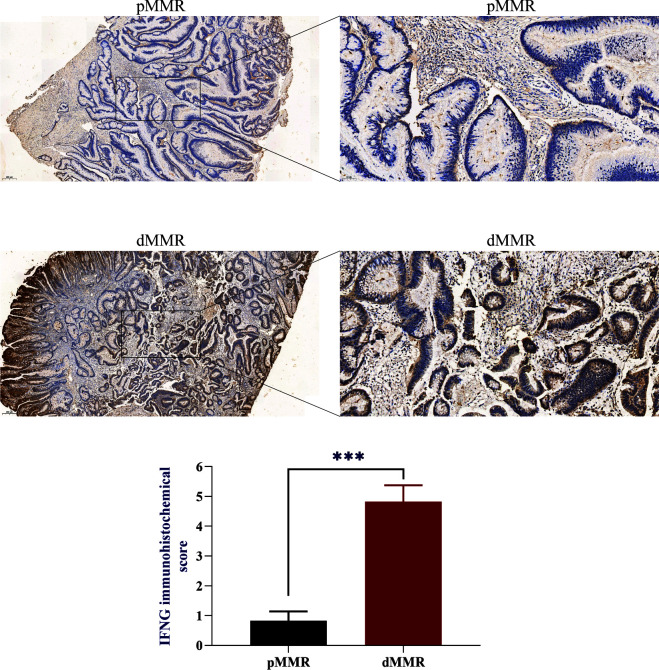
IFNG protein expression in pMMR and dMMR tissues. IHC staining showed that IFNG was significantly up-regulated in dMMR tissues compared with pMMR tissues. (***P-value <0.001).

To evaluate the IFNG mRNA expression in pMMR and dMMR COAD tissues, we isolated RNA from 10 pMMR tissues and 10 dMMR tissues and performed qRT-PCR. Compared with pMMR tissues, the expression of IFNG in dMMR was significantly increased (**Figure 6F**). For the protein expression of IFNG, we reached the same conclusion by performing immunohistochemical experiments on 5 pairs of pMMR and dMMR COAD tissues (**Figure 7**).

### Autophagy score and IFNG expression were significantly related to immune-related pathways

**Figure 8:**
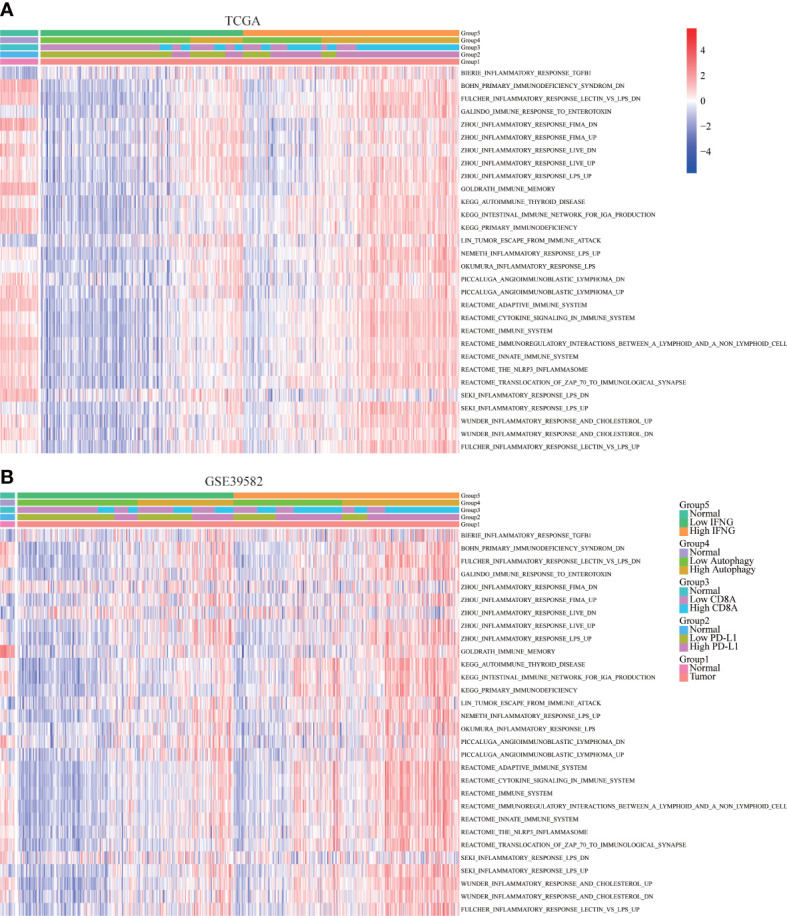
Correlation of immune-related pathways. In **(A)** TCGA and **(B)** GSE39582, the activity of most immune-related pathways in the high IFNG group and high autophagy score group were significantly higher.

To further determine the immune relevance, we explored the relationship between autophagy score, expression of IFNG, PD-L1 and CD8A and immune-related pathways. Heatmaps showed that most immune-related pathways were significantly and positively correlated with autophagy score and IFNG expression (**Figures 8A, B**).

### Autophagy score and IFNG expression were significantly related to immune signatures

**Figure 9:**
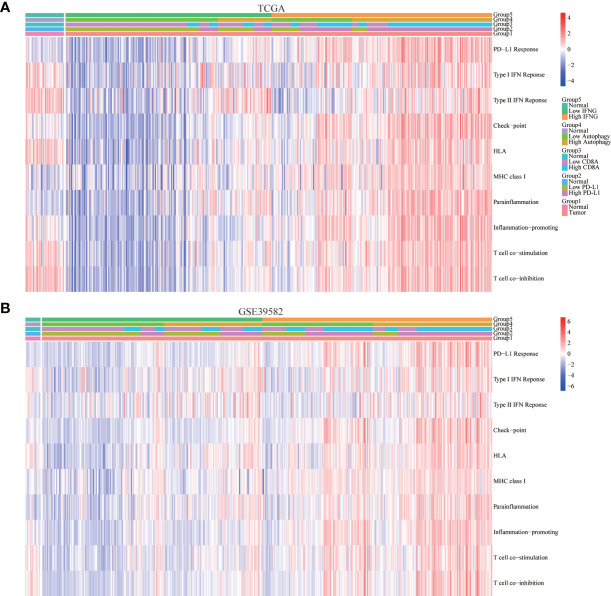
Correlation of immune signatures. In **(A)** TCGA and **(B)** GSE39582, the score of most immune signatures in the high IFNG group and high autophagy score group were significantly higher.

In addition to the above immune-related pathways, we obtained multiple gene sets of immune signatures from KEGG pathways and published articles (**Table S2**), including PD-L1 response, type II IFN response, type I IFN response, check-point reaction, HLA expression, para-inflammation, inflammation-promoting mechanism, T cell co-simulation, and T cell co-inhibition (37), whose activities were quantified using the ssGSEA algorithm. As shown in **Figures 9A, B**, similar to expression of PD-L1 and CD8A, autophagy score and expression of IFNG were significantly and positively correlated with the above immune signatures.

### Autophagy score and IFNG expression were significantly associated with concrete immune-related genes

**Figure 10:**
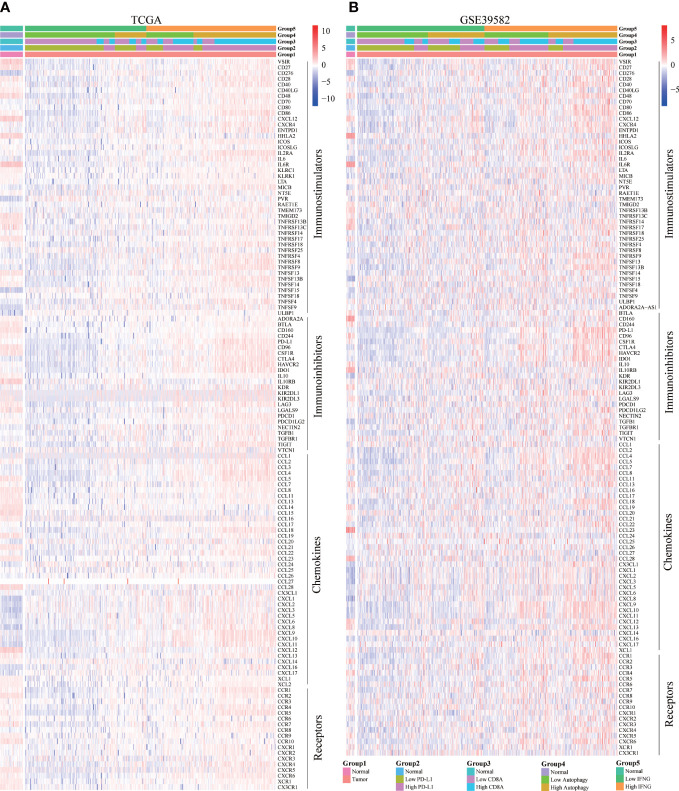
Correlation of concrete immune-related genes. In **(A)** TCGA and **(B)** GSE39582, the expression of most immune-related genes (immunostimulators, immunoinhibitors, chemokines, and chemokines receptors) in the high IFNG group and high autophagy score group were significantly higher.

Moreover, we explored the concrete correlation between autophagy score, expression of IFNG, PD-L1 and CD8A and immunostimulators, immunoinhibitors, chemokines, and chemokines receptors. As shown in **Figures 10A, B**, autophagy score, expression of IFNG, PD-L1 and CD8A were significantly and positively correlated with most immune-related genes.

### The landscape of somatic mutations in the low and high group of TCGA-COAD patients

**Figure 11:**
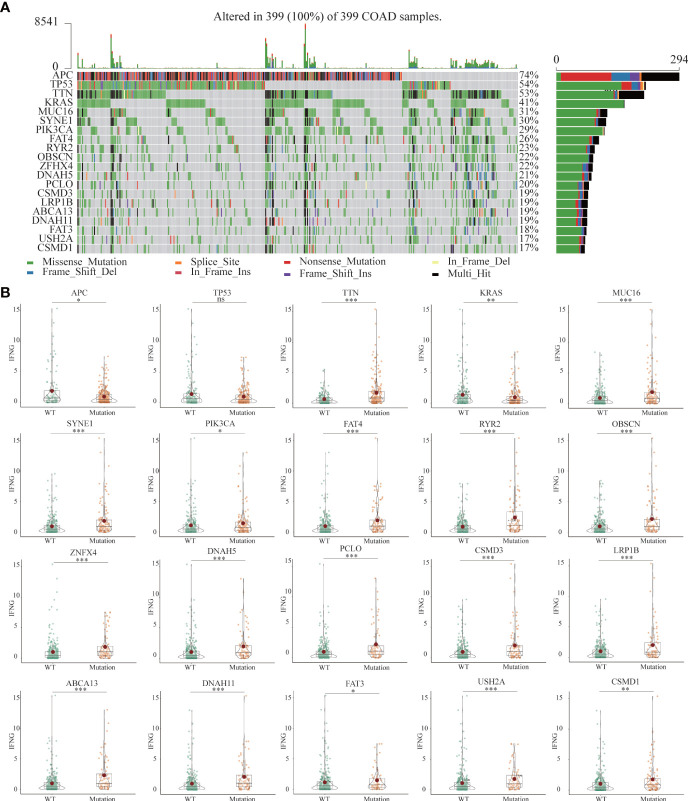
**(A)** The landscapes of somatic mutations of TCGA-COAD patients. **(B)** Among the top 20 mutant genes, differential expression of IFNG between wild-type (WT) and mutant tissues. (*P-value <0.05; **P-value <0.01; ***P-value <0.001).

Previous studies had shown that mutated tissues were suitable for immunotherapy (38). The MMR system participates in rectifying base-base mismatch, insertion, and deletion during DNA replication (39). If DNA damage is not repaired, it is possible to produce more mutations in somatic cells, which will change the sensitivity of tumor tissues to immunotherapy. **Figure 11A** showed the 20 most common mutated genes in COAD patients. Among the top 20 mutated genes, Except for APC, TTN and KRAS, the expression of IFNG was significantly increased in the other 16 mutant tissues (**Figure 11B**).

Based on the median values of autophagy score, IFNG, PD-L1 and CD8A expression, respectively, cases were classified into the low and high groups. Mutation information of each sample in the low and high groups was exhibited in waterfall plots. Among the top 20 mutated genes, apart from APC and TP53 mutations, COAD patients with high autophagy scores, IFNG, PD-L1 and CD8A expression had more mutation frequencies (**Figure S3**), which provided suggestions for clinical application of immunotherapy.

### Relevance of cisplatin chemotherapy

**Figure 12:**
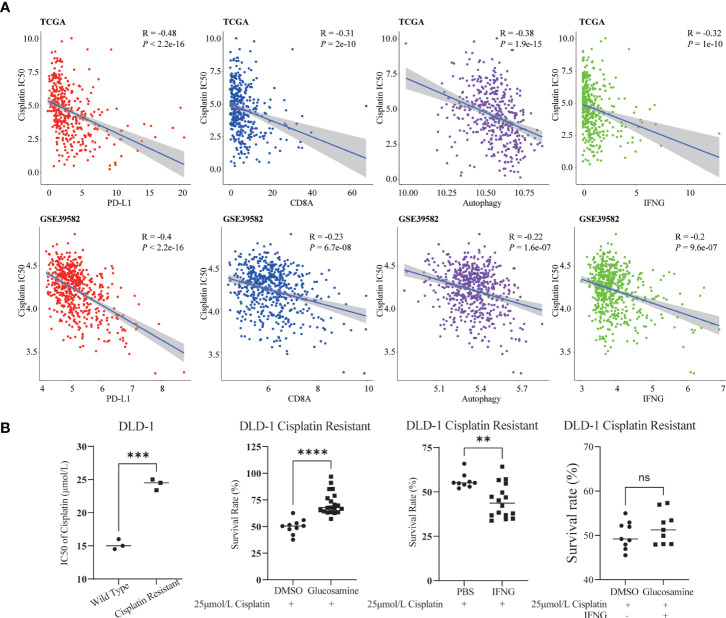
Relevance of cisplatin chemotherapy. **(A)** In two COAD cohorts, similar to PD-L1 and CD8A, autophagy score and IFNG were significantly correlated with cisplatin IC50. **(B)** Compared with wild type DLD-1, the cisplatin IC50 of drug resistant DLD-1 was significantly upregulated (***: P-value <0.001). In cisplatin resistant DLD-1, in combination with cisplatin IC50, glucosamine increased the survival of drug-resistant cells, while IFNG decreased the survival; After glucosamine administration, IFNG could reverse the increase of cell survival (**P-value <0.01; ****P-value <0.0001; ns, P-value >0.05).

Previous studies have shown that cisplatin augments antitumor T-cell responses leading to a potent therapeutic effect in combination with PD-L1 blockade (40, 41). Based on the Spearman correlation analysis, in TCGA and GSE39582, we concluded that similar to PD-L1 and CD8A, the autophagy score and IFNG were also significantly negatively correlated with the IC50 of cisplatin (**Figure 12A**).

In **Figure 12B**, compared with wild type DLD-1, the IC50 of cisplatin of drug resistant DLD-1 was significantly upregulated. To determine the effect of IFNG and its inhibitor glucosamine on the chemosensitivity of cisplatin in colon cancer, combined with the IC50 of cisplatin (25μmol/L), we examined the survival of cisplatin resistant DLD-1 after treatment with IFNG or glucosamine. CCK-8 assay showed that glucosamine increased cisplatin chemoresistance while IFNG increased cisplatin chemosensitivity. Furthermore, IFNG could reverse glucosamine induced chemoresistance.

### The gene set enrichment analysis

**Figure 13:**
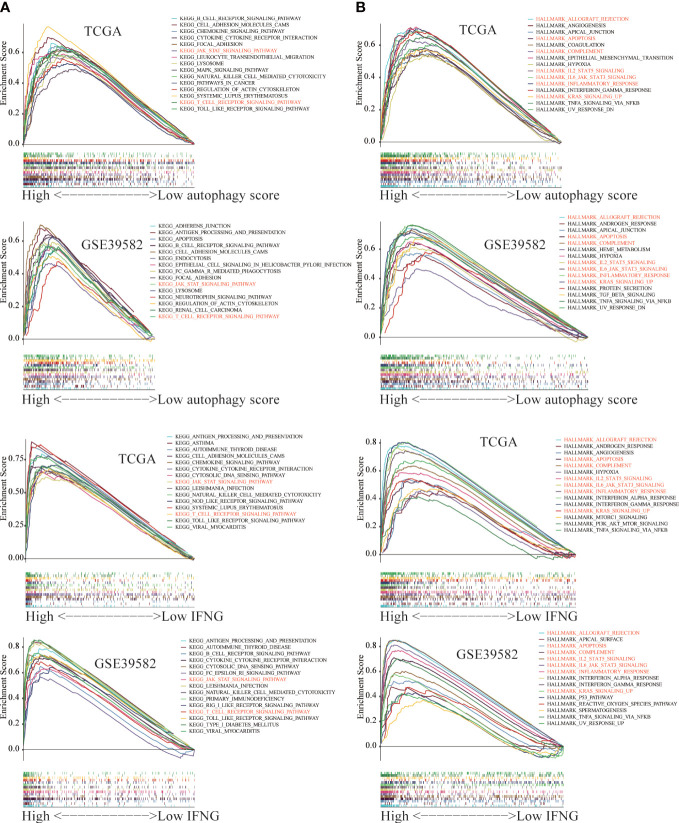
The Gene Set Enrichment Analysis (GSEA) between the high and low group of autophagy score and IFNG expression. The significant GSEA results shared by **(A)** TCGA and **(B)** GSE39582 were marked in red.

The GSEA was employed to identify key KEGG pathways and Hallmarks significantly correlated with autophagy score and IFNG expression. The top 15 significant gene sets enriched in high groups of autophagy scores, and IFNG expression were displayed in **Figure 13**. Among 186 KEGG pathways, high autophagy score and high IFNG expression were significantly related to KEGG_JAK_STAT_SIGNALING_PATHWAY and KEGG_T_CELL_RECEPTOR_SIGNALING_PATHWAY (**Figure 13A**). In 50 Hallmarks data sets, high autophagy score and high IFNG expression were significantly enriched in 7 Hallmarks, marked in red in **Figure 13B**. Combining shared GSEA results of KEGG pathways and Hallmarks, the JAK-STAT signaling pathway might play a vital role in the immunotherapy of COAD patients, which was consistent with previous research results (42).

### The ceRNA network and protein-protein interaction network

**Figure 14:**
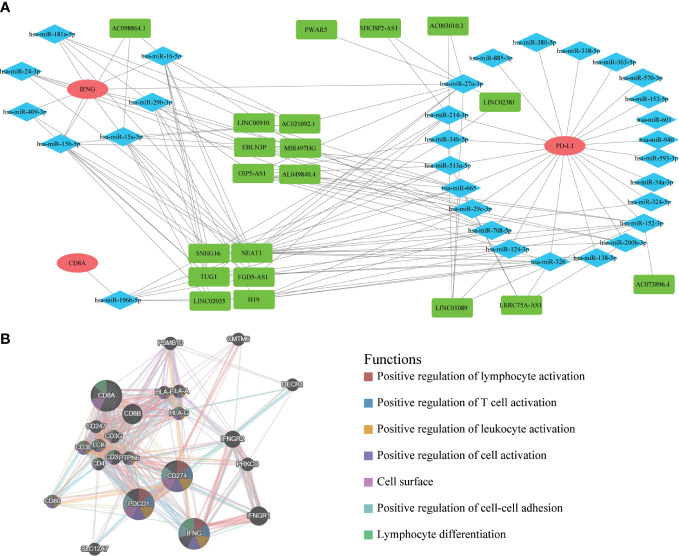
The ceRNA network and protein-protein interaction (PPI) network. **(A)** The ceRNA regulatory network of PD-L1, CD8A and IFNG. Red represented mRNAs; Blue represented miRNAs; Green represented lncRNAs. **(B)** The PPI network displayed 20 most relevant genes of PD-L1, CD8A, IFNG, and functional enrichment analysis of these 23 proteins.

After exploring the molecular pathway of IFNG, we began to mine the interacting molecules of IFNG. Previous studies had shown that long noncoding RNA (lncRNA) could act as microRNA (miRNA) sponges to regulate protein-coding gene (mRNA) expression. To explore the shared regulatory network of IFNG, PD-L1 and CD8A in predicting the efficacy of immunotherapy, we constructed the ceRNA regulatory network, including 19 lncRNAs, 31 miRNAs, and 3 mRNAs (**Figure 14A**). It should be noted that roles of 6 shared lncRNAs, SNHG16, NEAT1, TUG1, FGD5-AS1, LINC02035 and H19, in immunotherapy needed to be studied urgently. On the GeneMANIA website, we further developed a PPI network containing the 20 most relevant proteins with IFNG, PD-L1 and CD8A (**Figure 14B**). The functional enrichment results of these 23 proteins were also related to the activation of the immune system.

### Immune subtypes and molecular subtypes

**Figure 15:**
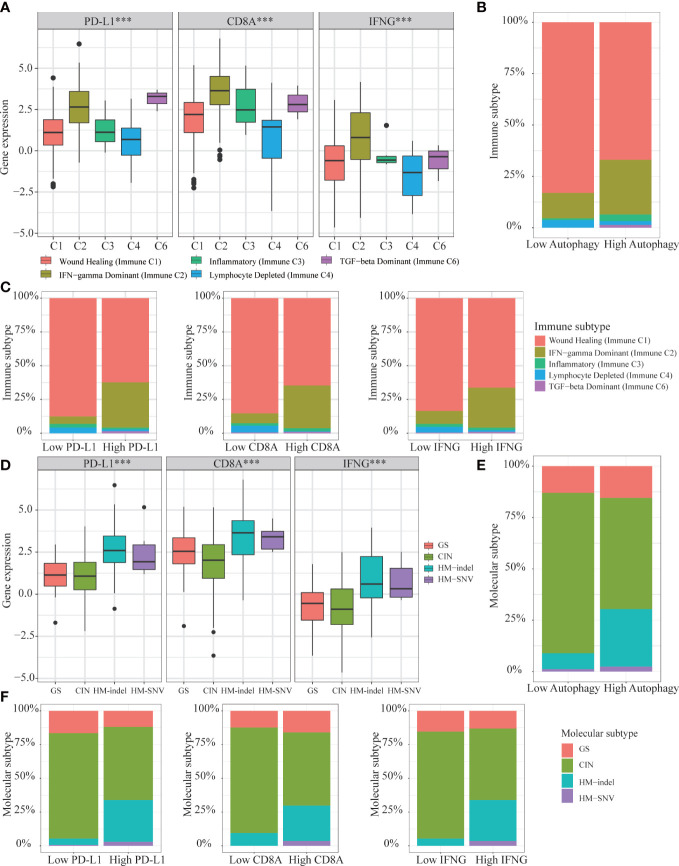
Correlation of autophagy score, IFNG, PD-L1 and CD8A expression with 5 immune subtypes and 4 molecular subtypes in TCGA-COAD. **(A)** In immune C2 and C6, expression of IFNG, PD-L1 and CD8A were consistent and higher. **(B, C)** In high group of autophagy score, PD-L1, CD8A, and IFNG expression, proportions of immune C2 and C6 were higher. **(D)** In HM-indel and HM-SNV, expression of IFNG, PD-L1 and CD8A were consistent and higher. **(E, F)** In high group of autophagy score, PD-L1, CD8A, and IFNG, proportions of HM-indel and HM-SNV were higher.

Based on above results, we concluded that autophagy score and IFNG expression could determine which COAD patients were suitable for immunotherapy. Then, we analyzed the expression of IFNG under existing clinical parameters. Combining the TCGA and GSE39582 data sets, we could only conclude that compared with stage IV patients, stage I patients had higher IFNG expression and were more suitable for immunotherapy (**Figure S4**). Regarding other clinical parameters, no unanimous conclusions were drawn. According to the global transcriptome, TCGA solid tumors were divided into 6 immune subtypes (C1-C6). Only 5 types of immune subtypes were identified in COAD queue, including wound healing (Immune C1), IFN-γ dominant (Immune C2), inflammatory (Immune C3), lymphocyte depleted (Immune C4), and TGF-β dominant (Immune C6) (43). Previous studies had confirmed that immune response correlated with somatic variation, while C2 and C6 were strongly correlated with somatic variation. As shown in **Figure 15A**, among 5 immune subtypes, immune C2 and C6 had higher expression of PD-L1, CD8A and IFNG. COAD patients with high autophagy score or high expression of PD-L1, CD8A and IFNG had a greater proportion of immune C2 and C6 (**Figures 15B, C**). Therefore, it was further confirmed that IFNG was as reliable biomarker as PD-L1 and CD8A. Besides, according to molecular characteristics, TCGA COAD consisted of 4 molecular subtypes: genome stable (GS), chromosomal instability (CIN), hypermutated- insertion and deletion (HM−indel) and hypermutated-single nucleotide variants (HM−SNV) (44). As shown in **Figure 15D**, among the 4 molecular subtypes, the HM-indel and HM-SNV had higher PD-L1, CD8A and IFNG. COAD patients with high autophagy score or high PD-L1, CD8A and IFNG had a greater proportion of HM-indel and HM-SNV (**Figures 15E, F**). We concluded that COAD patients of HM-indel and HM-SNV subtypes might benefit more from immunotherapy.

## Discussion

According to the latest guidelines, colon adenocarcinoma (COAD) is still a global problem threatening human health and life. Therefore, finding accurate prognostic and immunotherapeutic biomarkers will help solve the prognostic and treatment issues of COAD patients.

With the advent of the era of big data, large biological public databases, the Cancer Genome Atlas (TCGA) and Gene Expression Omnibus (GEO), are constantly being improved. Using bioinformatics methods to solve prognosis and treatment problems of COAD patient has attracted increasing attention from scientific researchers (45, 46).

Previous studies had confirmed that autophagy and its components participated in various immune responses, including innate immunity, inflammatory response and adaptive immunity (3, 47). In our research, we studied the prognosis and immunotherapy efficacy of COAD patients from the perspective of autophagy and further explored autophagy-related prognostic and immunotherapeutic biomarkers IFNG. Besides, IFNG might be a potential targeted therapy for cisplatin resistant colon cancer.

In addition to the antiviral activity, previous studies also showed IFNG had a dual pro-and anti-inflammatory effect in colon cancer (CC) (48). IFNG was produced by lymphocytes activated by specific antigens or mitogens (49). The IFNG-JAK-STAT-TET signaling pathway, which mediated anti-PD-L1/PD-1 immunotherapy, was frequently disrupted in solid tumors (42). This was consistent with our analysis, and the function of IFNG was significantly related to the JAK-STAT signaling pathway.

Besides, IFNG was also a key mediator of antitumor immunity with angiostatic activity, mediated the effects of anti-CTLA4 therapy on vessel perfusion and tumor growth (50). IFNG was critical for vessel modulation after immunotherapy blockade (51). At the same time, it was also an effective activator of macrophages and could enhance the antiviral and antitumor effects mediated by type I interferon (52). In CC, specific roles and more mechanisms of IFNG in immunotherapy needed to be studied.

From the perspectives of the immune score, stromal score, tumor purity, tumor-infiltrating immune cell abundance, immune regulators, immune-related pathways, immune signatures, tumor mutational burden (TMB), microsatellite instability (MSI), mismatch repair (MMR) ability, the proportion of somatic mutations and subtype analysis, we confirmed that COAD patients with high autophagy score or high IFNG expression would benefit more from immune blocking therapy. In terms of prognosis, it was equally important to accurately predict the overall survival (OS) and disease-free survival (RFS). It was worth noting that the RFS of the high IFNG group was significantly longer than that of the corresponding low group.

By constructing the ceRNA network of 3 mRNA (IFNG, PD-L1 and CD8A), we obtained 6 shared lncRNAs, including SNHG16, NEAT1, TUG1, FGD5-AS1, LINC02035 and H19. Among them, there had been some research on the relationships between FGD5-AS1, H19 and PD-L1 (53–55). The relationship between SNHG16, NEAT1, TUG1, FGD5-AS1, LINC02035 and PD-L1 needed further research.

Previous studies had proved the JAK-STAT signaling pathway was involved in the regulation of the immune function of colon cancer and rectal cancer (56). In terms of molecular mechanisms, we conducted GSEA and found that KEGG_JAK_STAT_SIGNALING_PATHWAY and HALLMARK_IL6_JAK_STAT3_SIGNALING were significantly enriched in the high autophagy group and high IFNG group. In other words, the above 2 pathways were highly enriched in COAD patients who benefited more from immunotherapy.

More and more studies have found that there is a synergy between chemotherapy and immunotherapy (57). In our research, through correlation analysis, we further proved the link between autophagy and IFNG and chemotherapy.

The advantage of this study was that 2 data sets, TCGA and GSE39582, supported our research and conclusions. The RFS and OS, two prognostic indicators, were included in our survival analysis. Besides, we demonstrated the predictive effect of IFNG level on the efficacy of immunotherapy or cisplatin chemotherapy from multiple perspectives, including wet experiments.

## Conclusions

Autophagy-related IFNG could simultaneously predict the RFS and the efficacy of immune blockade of COAD patients. Autophagy score was an effective immunotherapeutic biomarker, but not a prognostic indicator for COAD patients. IFNG might be a potential targeted therapy for cisplatin resistant colon cancer. HM-indel and HM-SNV subtypes might benefit more from immunotherapy.

## Data availability statement

The original contributions presented in the study are included in the article/**Supplementary Material**. Further inquiries can be directed to the corresponding authors.

## Ethics statement

The studies involving human participants were reviewed and approved by Peking University First Hospital Biomedical Research Ethics Committee. The patients/participants provided their written informed consent to participate in this study.

## Author contributions

This research was designed by LR and PW. The manuscript was written by TY. The data analysis was performed by TY, YL and SC. The surgical specimens used in this article was collected by TY. The *in vitro* experimental verification was done by TY, YL, YC and JZ. All authors contributed to the article and approved the submitted version.
