# Supplementary figures and images for: Autophagy-related IFNG is a prognostic and immunochemotherapeutic biomarker of COAD patients
Source: Front Immunol. 2023 Jan 23;14:1064704. doi: 10.3389/fimmu.2023.1064704 (PMC9900120; doi:10.3389/fimmu.2023.1064704)

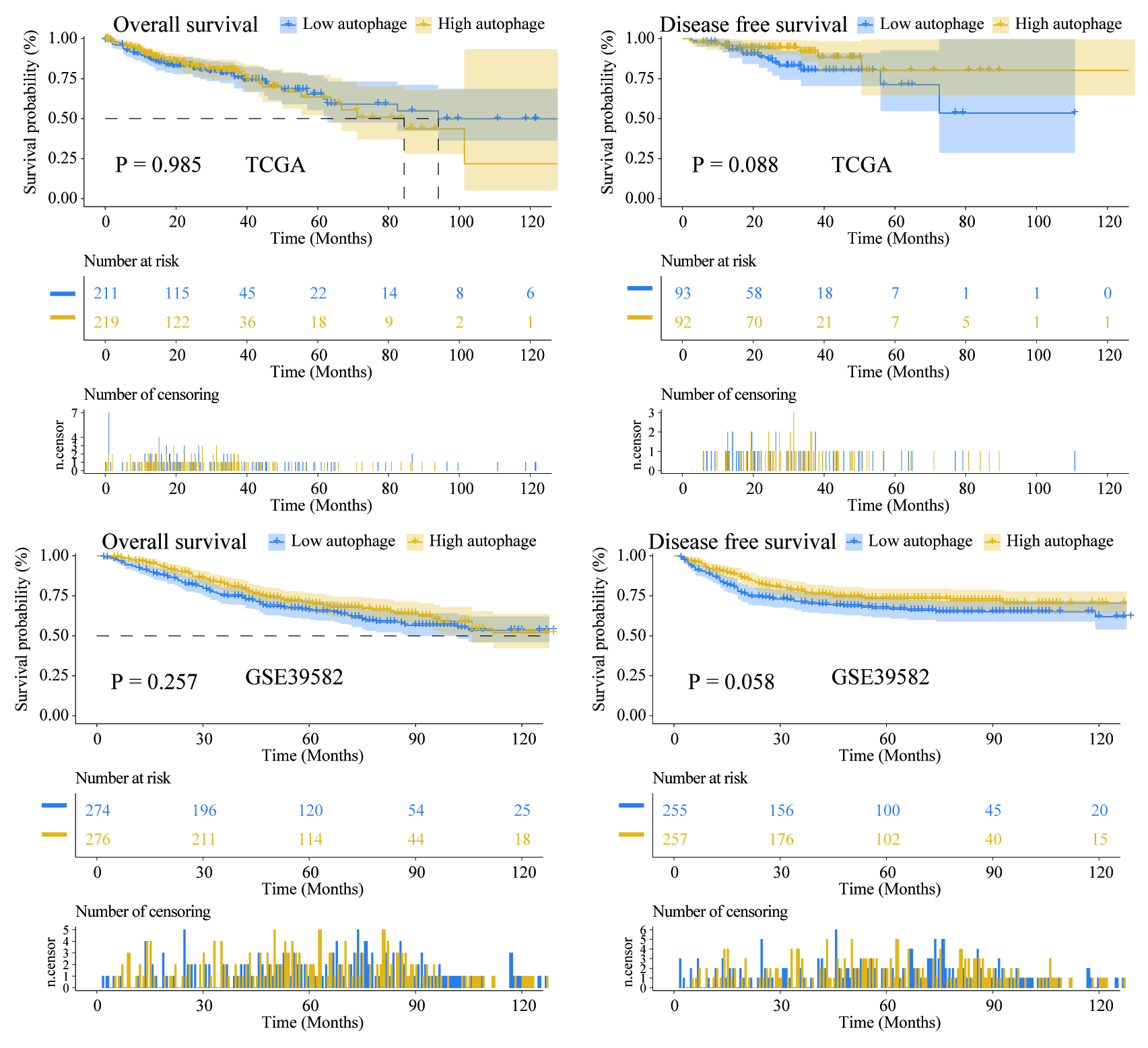

Supplement: Supplementary file 1 [file Image_1.tif]

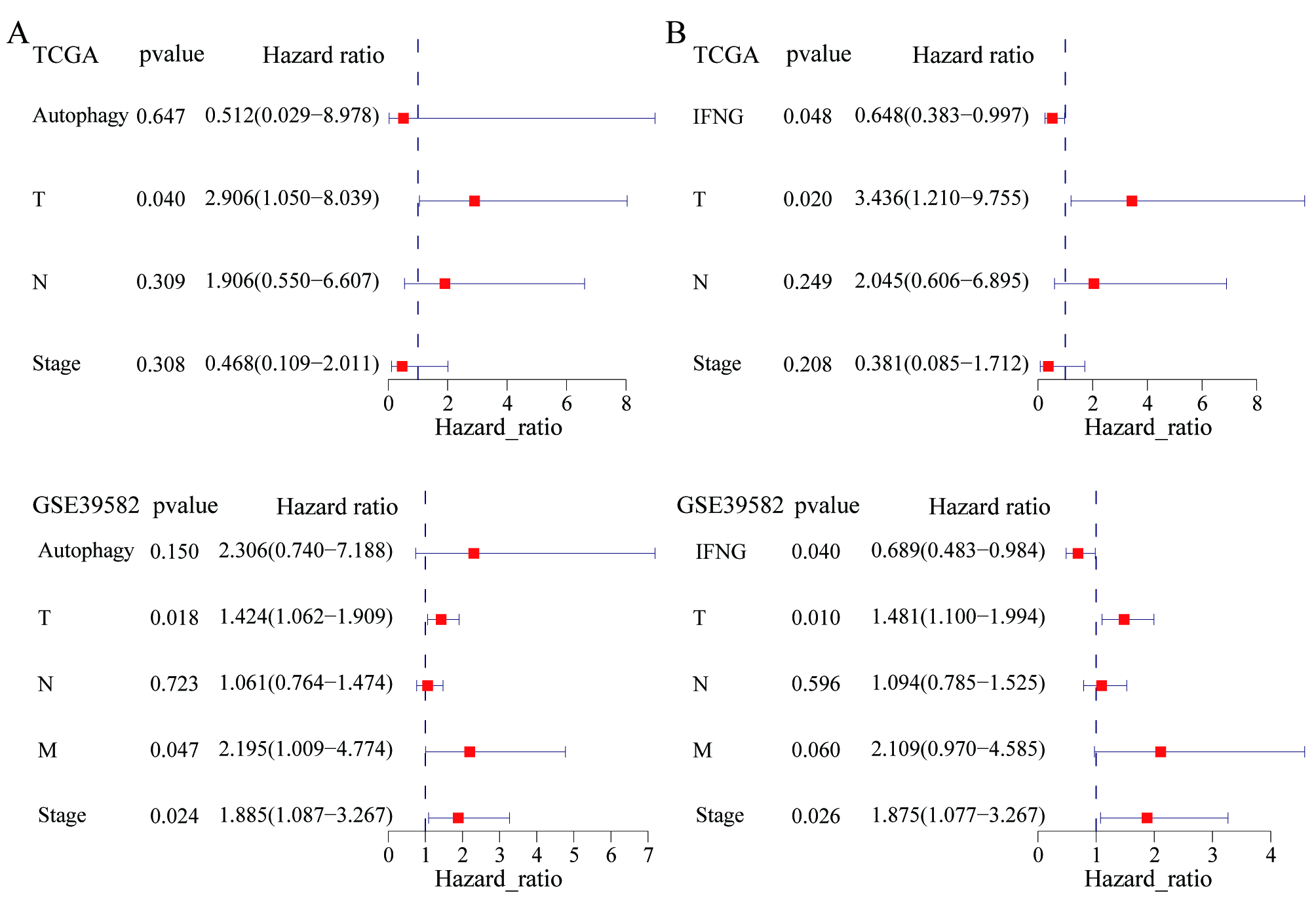

Supplement: Supplementary file 2 [file Image_2.tif]

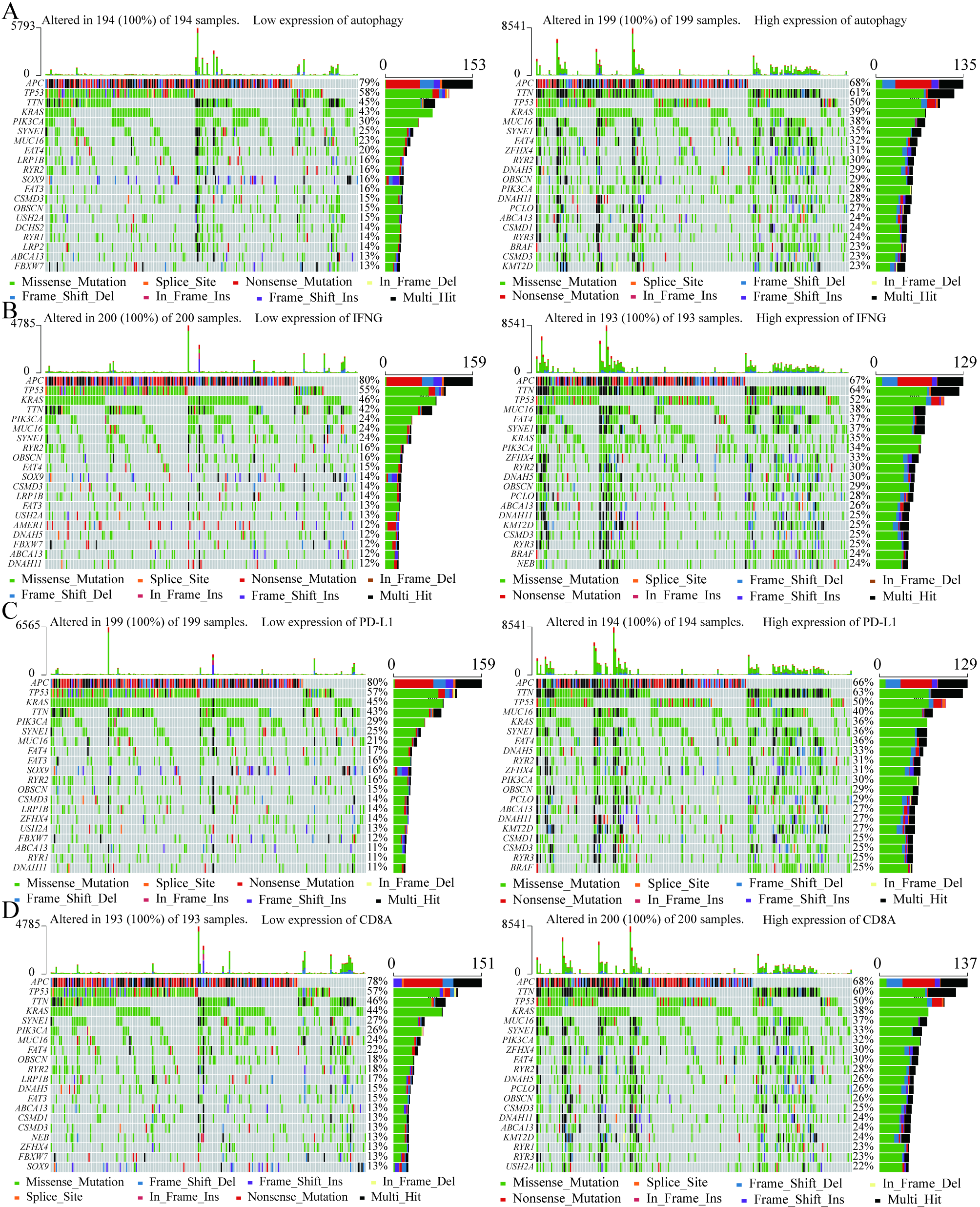

Supplement: Supplementary file 3 [file Image_3.tif]

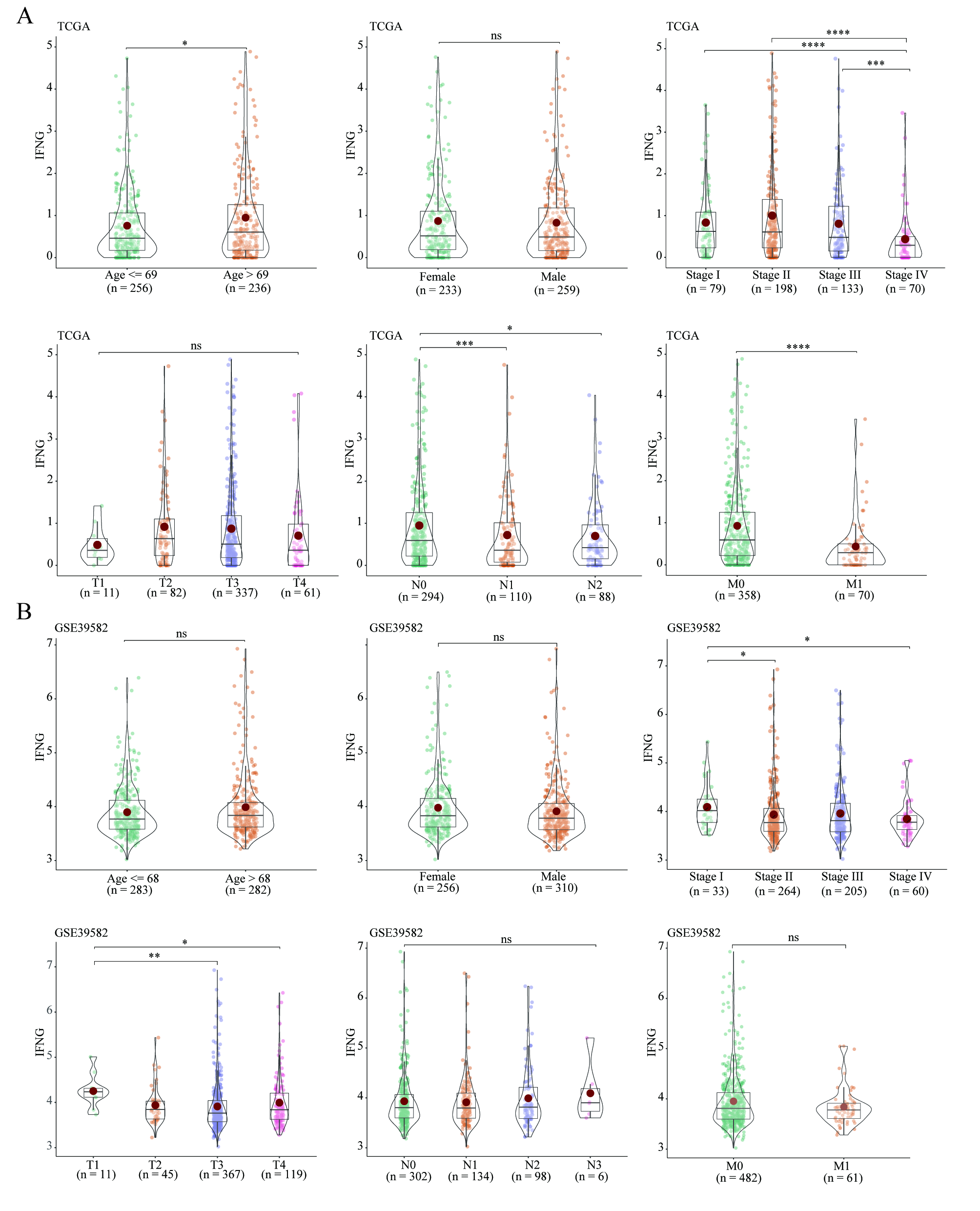

Supplement: Supplementary file 4 [file Image_4.tif]
