# Supplementary material for: Autophagy-related IFNG is a prognostic and immunochemotherapeutic biomarker of COAD patients
Source: Front Immunol. 2023 Jan 23;14:1064704. doi: 10.3389/fimmu.2023.1064704 (PMC9900120; doi:10.3389/fimmu.2023.1064704)
Supplement: Supplementary file 5 [file Table_1.docx]

**Table S1**. 232 autophagy-related genes (ARGs) obtained from the Human Autophagy Database (HADb) in our study.

| Autophagy-related genes (ARGs) | | | | | | | | |
| --- | --- | --- | --- | --- | --- | --- | --- | --- |
| AMBRA1 | APOL1 | ARNT | ARSA | ARSB | ATF4 | ATF6 | ATG10 | ATG12 |
| ATG16L1 | ATG16L2 | ATG2A | ATG2B | ATG3 | ATG4A | ATG4B | ATG4C | ATG4D |
| ATG5 | ATG7 | ATG9A | ATG9B | ATIC | BAG1 | BAG3 | BAK1 | BAX |
| BCL2 | BCL2L1 | BECN1 | BID | BIRC5 | BIRC6 | BNIP1 | BNIP3 | BNIP3L |
| C12orf44 | C17orf88 | CALCOCO2 | CAMKK2 | CANX | CAPN1 | CAPN10 | CAPN2 | CAPNS1 |
| CASP1 | CASP3 | CASP4 | CASP8 | CCL2 | CCR2 | CD46 | CDKN1A | CDKN1B |
| CDKN2A | CFLAR | CHMP2B | CHMP4B | CLN3 | CTSB | CTSD | CTSL1 | CX3CL1 |
| CXCR4 | DAPK1 | DAPK2 | DDIT3 | DIRAS3 | DLC1 | DNAJB1 | DNAJB9 | DRAM1 |
| EDEM1 | EEF2 | EEF2K | EGFR | EIF2AK2 | EIF2AK3 | EIF2S1 | EIF4EBP1 | EIF4G1 |
| ERBB2 | ERN1 | ERO1L | FADD | FAM48A | FAS | FKBP1A | FKBP1B | FOS |
| FOXO1 | FOXO3 | GAA | GABARAP | GABARAPL1 | GABARAPL2 | GAPDH | GNAI3 | GNB2L1 |
| GOPC | GRID1 | GRID2 | HDAC1 | HDAC6 | HGS | HIF1A | HSP90AB1 | HSPA5 |
| HSPA8 | HSPB8 | IFNG | IKBKB | IKBKE | IL24 | IRGM | ITGA3 | ITGA6 |
| ITGB1 | ITGB4 | ITPR1 | GAA | GABARAP | GABARAPL1 | GABARAPL2 | GAPDH | GNAI3 |
| GNB2L1 | GOPC | GRID1 | GRID2 | KIAA0226 | KIAA0652 | KIAA0831 | KIF5B | KLHL24 |
| LAMP1 | LAMP2 | MAP1LC3A | MAP1LC3B | MAP1LC3C | MAP2K7 | MAPK1 | MAPK3 | MAPK8 |
| MAPK8IP1 | MAPK9 | MBTPS2 | MLST8 | MTMR14 | MTOR | MYC | NAF1 | NAMPT |
| NBR1 | NCKAP1 | NFE2L2 | NFKB1 | NKX2-3 | NLRC4 | NPC1 | NRG1 | NRG2 |
| NRG3 | P4HB | PARK2 | PARP1 | PEA15 | PELP1 | PEX14 | PEX3 | PIK3C3 |
| PIK3R4 | PINK1 | PPP1R15A | PRKAB1 | PRKAR1A | PRKCD | PRKCQ | PTEN | PTK6 |
| RAB11A | RAB1A | RAB24 | RAB33B | RAB5A | RAB7A | RAC1 | RAF1 | RB1 |
| RB1CC1 | RELA | RGS19 | RHEB | RPS6KB1 | RPTOR | SAR1A | SERPINA1 | SESN2 |
| SH3GLB1 | SIRT1 | SIRT2 | SPHK1 | SPNS1 | SQSTM1 | ST13 | STK11 | TBK1 |
| TM9SF1 | TMEM49 | TMEM74 | TNFSF10 | TP53 | TP53INP2 | TP63 | TP73 | TSC1 |
| TSC2 | TUSC1 | ULK1 | ULK2 | ULK3 | USP10 | UVRAG | VAMP3 | VAMP7 |
| VEGFA | WDFY3 | WDR45 | WDR45L | WIPI1 | WIPI2 | ZFYVE1 |  |  |
